# Activation of Brown Adipocytes by Farnesoid X Receptor Agonist, Obeticholic Acid—A Potential Novel Therapeutic Avenue in the Management of Obesity

**DOI:** 10.3390/jcm15083081

**Published:** 2026-04-17

**Authors:** Anna Maria Dąbrowska, Mirosława Chwil, Ewa M. Urbańska

**Affiliations:** 1 Department of Experimental and Clinical Pharmacology, Medical University of Lublin, Jaczewskiego Street 8b, 20-090 Lublin, Poland; 2 Department of Botany and Plant Physiology, University of Life Sciences in Lublin, Akademicka 15, 20-950 Lublin, Poland; miroslawa.chwil@up.lublin.pl

**Keywords:** obesity, bile acids, obeticholic acid, brown adipose tissue, farnesoid X receptor

## Abstract

Obesity, a heterogeneous metabolic disease, is linked with severe comorbidities, prominently increasing morbidity and mortality. A weight loss between 5% and 10% is already sufficient to induce clinically relevant improvements in human health. Activation of energy expenditure through an impact on the brown and beige adipose tissues has recently become an interesting new target in obesity treatment. Obeticholic acid (OCA) is a semisynthetic derivative of the primary human bile acid, chenodeoxycholic acid. The compound is an agonist of farnesoid X receptor (FXR) and Takeda G protein-coupled receptor (TGR5), activating the cellular pathways such as fibroblast growth factor-19, tissue-specific uncoupling protein 1, or type 2 iodothyronine deiodinase associated with energy expenditure and brown adipose tissue activity. So far, OCA has been approved to treat primary biliary cholangitis. Interestingly, the drug demonstrated therapeutic effects in animal models of obesity. Preliminary results from the human studies show that OCA administration holds potential as a treatment option in obesity, although some adverse effects may occur. Long-term administration of OCA might constitute an attractive therapeutic add-on approach, complementary to the currently approved treatments. The design of OCA derivatives targeting similar mechanisms, yet with a better pharmacological profile, seems to be an exciting pathway in the search of novel anti-obesity drugs. Further clinical trials involving larger cohorts of patients, with and without comorbidities, are warranted to confirm the benefits and safety of OCA administration.

## 1. Introduction

Maintaining a body weight appropriate for one’s age, lifestyle, and health conditions is a key aspect of disease prevention and management. Genetic, epigenetic, and environmental factors alter the gut–brain axis, modify the immune and autonomic responses, and impact the balance between individual energy needs, food intake, and physical activity [1,2]. In consequence, eating disorders and obesity are nowadays considered multifactorial conditions of neurometabolic origin and constitute a significant therapeutic challenge [3,4,5]. Currently, the prevalence of obesity has reached the dimension of a global health epidemic. It is estimated that above 1 billion adults suffer from either overweight or obesity, and the number of affected individuals is predicted to rise dramatically among children and adolescents [2]. Although the definition of obesity is debated, the criterion BMI > 30 kg/m^2^ is often used as a screening tool. Obesity is a chronic condition characterized by excessive long-term energy intake that exceeds individual energy expenditure [1,2]. Due to its metabolic consequences, it is an established risk factor for conditions such as type 2 diabetes, steatohepatitis, osteoarthritis, and cardiovascular disease, imposing a significant social and economic burden [2,6].

Therapeutic interventions are based on three modalities: lifestyle intervention, pharmacotherapy, and weight-loss procedures, including bariatric surgery [6]. In recent years, exciting advances have occurred in the field of pharmacotherapy. The introduction of novel drugs impacting the incretin and amylin pathways has transformed clinical management; however, high costs, weight regain post-discontinuation, and adverse events are notable limitations [6]. Thus, there is an ongoing need to explore different strategies for weight reduction. However, several novel compounds tested clinically failed to produce a substantial weight reduction or exhibited a poor safety profile.

An increase in energy expenditure might be either an alternative or an additional option to diet and exercise, the currently approved therapies [6,7]. However, no drug with a direct effect on the increase in energy expenditure through the influence on adipose tissue has been available so far. In humans, there are two kinds of adipose tissue with distinct physiologic functions: white adipose tissue and brown adipose tissue (BAT) [8,9]. Classical white adipocytes specialize in the storage of excessive triglycerides when energy intake exceeds energy expenditure. BAT and related beige adipocytes play a central role in metabolizing glucose and fatty acids to produce heat through activation of the thermogenic tissue-specific uncoupling protein 1 (UCP1) [8,9]. It has been calculated that a permanent 5% increase in resting metabolic rate could have major effects on body weight regulation and metabolic health [10]. Although the data on BAT activation are indirect and derived from in vitro or rodent studies, and human evidence is still limited, the activation of energy expenditure through an impact on the brown and beige adipose tissues has recently become an interesting new target in obesity treatment [11].

Obeticholic acid (OCA; INT-747), an orally available, hydrophobic derivative of chenodeoxycholic acid (6-alpha-ethyl-chenodeoxycholic acid), has been developed for the treatment of biliary cholangitis and was approved in the USA and in the EU in 2016 [12,13]. Recent data indicate that OCA may activate BAT, leading to an increase in energy expenditure and ameliorating obesity [12,13]. Here, we provide an update on the potential role of OCA in the management of metabolic disease and discuss its role as a novel therapeutic agent to treat obesity.

## 2. BAT as a Novel Therapeutic Target to Treat Obesity

The presence of BAT in the neonate human body and its role in heat production were described approximately six decades ago. Subsequently, its potential role in the regulation of energy expenditure has been postulated in adults [14,15]. Larger quantities of brown adipocytes were detected mainly in the supraclavicular fossa, subclavian area, and axilla, with fewer groups in mediastinal, paraspinal, perinephric, and supradrenal areas [16,17].

Non-shivering thermogenesis is mediated through stimulation of UCP1 on the inner mitochondrial membrane within brown adipocytes. Subsequent uncoupling of mitochondrial respiration by dissociating electron transport from ATP production leads to heat production [18,19]. A similar function was ascribed to specific white adipocytes, called beige cells, located predominantly in white adipose tissue and undergoing so-called browning [14]. The process of white adipocyte browning is linked to genomic reprogramming resulting in an increased number of mitochondria and higher expression of genes contributing to energy burning, e.g., peroxisome proliferator-activated receptor-alpha and gamma (PPARα and PPARγ), or UCP1 [10,20]. Brown and beige adipocytes may be functional in adults; however, they occur in rather small quantities. Thus, energy metabolism may be affected by stimulating the activity of brown adipocytes or by increasing beige fat cell formation [8,10,12].

Previous studies have confirmed that bile acids and their analogs increase BAT mass and BAT activity, which may lead to a decrease in diet-induced obesity [11,21]. Bile acids are essential factors in lipid metabolism, being the most important route for elimination of cholesterol from the circulation [11]. After their secretion into the intestine, about 95% of bile acids are reabsorbed from the terminal ileum, entering enterohepatic circulation [22]. The remaining 5% is transformed into secondary bile acids by intestinal microorganisms [23]. Gut microbiota contribute to the modulation of cholesterol metabolism through nuclear farnesoid X receptor (FXR)-dependent regulation of the expression of fibroblast growth factor 19 (FGF-19) in the ileum and *CYP7A1* and *CYP8B1* in the liver [23].

Despite the data implying FXR-mediated BAT activation, its quantitative contribution to total energy expenditure in adult humans remains not fully clarified. While preclinical models demonstrate robust UCP1-dependent thermogenesis, human BAT depots are significantly smaller and more dispersed. Current assessments relying on 18F-FDG PET/CT may overestimate metabolic activity due to methodological limitations in capturing chronic, low-level thermogenesis. Furthermore, the reported ~5% increase in energy expenditure following bile acid-mediated activation may be not sufficient to induce clinically significant weight loss. However, the metabolic benefit of activated BAT may be qualitative, increasing glucose and free fatty acid metabolism, thereby improving systemic insulin sensitivity rather than inducing profound caloric deficit.

## 3. Targets of Bile Acids

### 3.1. FXR Signaling

FXR (also termed NRIH4) belongs to a family of nuclear hormone receptors. Initially considered an orphan receptor, FXR was cloned in 1995 [24,25]. It is activated by farnesol derivatives, which are metabolic intermediates of the mevalonate pathway [26]. In humans, the gene for FXR (*NR1H4*) is localized on chromosome 12q23.1. Four different isoforms have been identified (FXRα1, FXRα2, FXRα3, and FXRα4) [23,27]. FXR may dimerize with retinoid receptor or bind to its DNA response element in the promoter region of a gene, leading to the transcription of target genes [26,28]. FXR is highly expressed in the liver and in the intestines, and at lower levels in the kidneys, adipose tissue, or the adrenal glands [26].

Over the past decade, a large group of FXR agonists has been described, including endogenous ligands (bile acids), semi-synthetic bile acids, such as OCA, and entirely synthetic agonists (e.g., fexaramine, GW4064, PX-102, XL335, GSK2324) [29]. Primary bile acids cholic acid (CA) and chenodeoxycholic acid (CDCA), secondary bile acids deoxycholic acid (DCA) and lithocholic acid (LCA), as well as taurine or glycine conjugates were identified as FXR endogenous agonists. The potency of natural bile acids to activate FXR was CDCA > DCA > LCA > CA [23,26]. Activation of FXR in enterocytes by bile acids stimulates the transcription of various genes, leading to the suppression of de novo synthesis of bile acids from cholesterol, through the release of FGF-19 into the hepatic portal circulation. It also increases the transport of bile acids out of hepatocytes, thus reducing hepatic injury [13,30]. FGF-19 is a secreted peptide, released postprandially from the small intestine. In humans, FGF-19 plasma concentration reaches a peak at 2–3 h after a meal [31,32,33,34]. FGF-19 binds to the Fibroblast Growth Factor Receptor 4 (FGFR-4) on the hepatocytes, which leads to inhibition of the expression of the *CYP7A1* gene and *CYP8B1* gene. As a result, the activity of cholesterol 7α-hydroxylase (*CYP7A1*), the enzyme responsible for converting cholesterol into bile acids, is suppressed, and hepatic bile acid synthesis is inhibited. The repression of *CYP8B1*, a sterol 12-a hydroxylase, is key to regulating the ratio of CA to CDCA during bile acid synthesis [22,23]. The FXR/FGF-19/FGFR-4 pathway is a major negative regulator of bile acid synthesis [22].

Recent findings highlight the therapeutic potential of FXR-targeted pharmacological interventions in addressing obesity and its related metabolic complications. Experimental research data indicate that FGF-19 and its mouse ortholog FGF-15 are activators of the thermogenic program in BAT. In FGF-15 transgenic mice, the BAT mass and thermogenesis increase [35]. Transgenic mice overexpressing FXR have elevated levels of FGF-15 and are protected from metabolic complications of a high-fat diet, such as weight gain, glucose intolerance, and altered lipid profile [36,37].

Interestingly, FGF-15 increases metabolic rate without a significant change in food intake. In BAT, chronic exposure to FGF-15 results in a higher gene expression profile that is consistent with activation of this tissue, especially of genes that control glycolysis, fatty acid metabolism, and mitochondrial function [31,32,35]. FXR activation promotes insulin sensitivity, decreases circulating triglycerides, and reduces hepatic gluconeogenesis and glycogenolysis [13]. Furthermore, FXR plays an essential role in the modulation of the intestinal flora metabolism and may impact oxidative stress and inflammation [28,31,32].

In contrast, it was revealed that overexpression of another member of the FGF family, FGF-16, in mice results in prominent weight loss, not due to increased energy expenditure, but as a consequence of an impaired feeding pattern [38]. Furthermore, it was recently shown that the genetic knockout of intestinal FXR actually attenuates the development of obesity in response to a high-fat diet [39]. Therefore, the regulatory role of FXR in energy homeostasis is nuanced and cannot be reduced to a simple anti-obesity paradigm. The overall outcome of FXR stimulation is influenced by the target organ, the specific ligand employed, and the presence of comorbidities. In general, the intestine-specific change of FXR activity is associated with beneficial metabolic modifications [40].

### 3.2. TGR5 Pathway

Takeda G protein-coupled receptor (TGR5), also known as G protein-coupled bile acid receptor 1 (GPBAR1) or membrane-type receptor for bile acids (MBAR), belongs to a G protein-coupled receptor (GPCR) family. It is widely expressed in the intestinal epithelial cells and hepatocytes and, in contrast to FXR, is activated primarily by secondary bile acids [41]. TGR5, through the cAMP-mediated pathway, impacts proliferation, metabolism, and inflammation [41]. Furthermore, it was demonstrated that TGR5 activation promotes energy expenditure in thermogenic tissues through the induction of type 2 iodothyronine deiodinase (D2) and the uncoupling of protein 1 (UCP1) [34,42]. In BAT and skeletal muscle cells, stimulation of cAMP-dependent protein kinase A activates cAMP response element binding protein (CREB) and induces D2 and UCP1 [43,44,45,46]. D2-mediated conversion of thyroxine to active T3 increases BAT activity and energy expenditure, ultimately resulting in weight loss and enhanced insulin sensitivity [11,43]. Moreover, D2 and UCP1, as browning factors, may stimulate energy metabolism in white adipose tissue [44,45,46].

Under various experimental conditions, activation of TGR5 signaling was able to mitigate obesity by reducing caloric intake, increasing energy expenditure via BAT and muscle thermogenesis [47]. In the mouse model of diet-induced obesity, hypothalamic knockout of TGR5 led to hyperphagia and obesity [48]. In line with the above, the use of a mixture of bile acids reduced food intake and body mass, whereas TGR5 deletion induced obesity in mice [49]. Clinical data revealed that TGR5 expression in human adipose tissue correlates positively with obesity, whereas it is decreased during diet-induced weight loss [50]. TGR5 has also been reported to enhance insulin secretion through enhanced release of glucagon-like peptide-1 (GLP-1), and subsequent improvement of glucose homeostasis [43,51,52]. Thus, TGR5-mediated enhancement of energy use in BAT seems an important aspect of bile acids-induced reduction of body weight.

## 4. Bile Acids, OCA, and Weight Loss

### 4.1. Animal Studies

**Figure 1:**
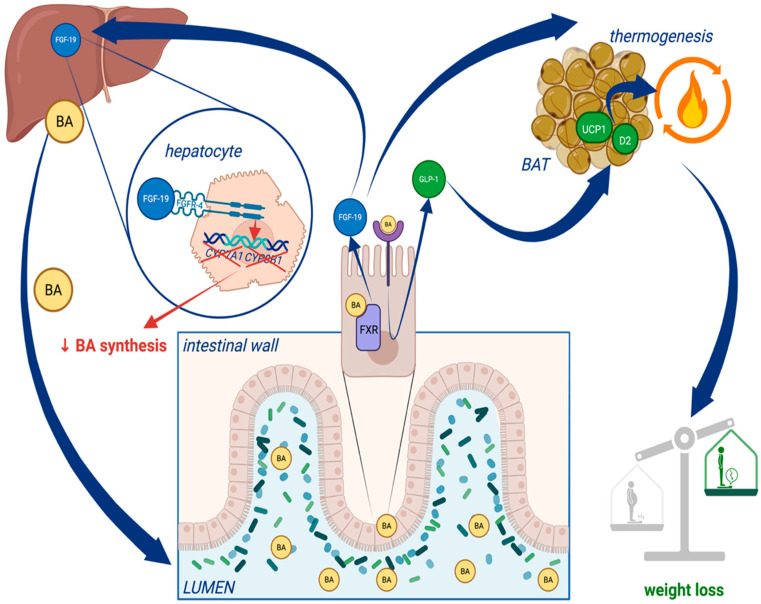
The role of bile acids in the regulation of metabolism and weight. BAT—brown adipose tissue, BA—bile acid, *CYP7A1*—cytochrome P450 family 7 subfamily A member 1 gene, *CYP8B1*—cytochrome P450 family 8 subfamily B member 1 gene, D2—type 2 iodothyronine deiodinase, GI—gastrointestinal system, GLP-1—glucagon-like peptide-1, FGF-19—fibroblast growth factor 19, FGFR-4—fibroblast growth factor receptor 4, FXR—farnesoid X receptor, UCP1—uncoupling protein 1. Created in BioRender. Urbanska, E. (2026) https://BioRender.com/gk7254w.

Administration of bile acids, as natural FXR and TGR5 agonists, as well as augmentation of FXR- and TGR5-related cellular pathways, may increase whole-body energy expenditure, preventing obesity and insulin resistance. Indeed, such effects were observed under experimental conditions in various animal studies [31,32,35,44,46]. Recombinant FGF19 (1 mg/kg i.v., for 7 days) increased metabolic rate, reduced body weight, and reversed the diabetes in both high-fat-fed mice and leptin-deficient mice [31]. Injection of FGF19 into the lateral ventricle of the brain also increased metabolic rate [31]. Administration of FGF21 at 0.1–10.0 mg/kg for 6 weeks increased energy expenditure and reduced body weight in the mouse model of diet-induced obesity [53] (Figure 1).

In FGF19 transgenic mice, a significant and specific reduction in fat mass that resulted from an increase in energy expenditure was observed [35]. Mice had increased brown adipose tissue mass, decreased liver expression of acetyl coenzyme A carboxylase 2, and lower hepatic triglyceride content [35]. In transgenic mice receiving a high-fat diet, TGR5 overexpression significantly improved glucose tolerance in obese mice [46]. However, no change in their weight was observed, despite increased energy expenditure [46]. Nevertheless, administration of TGR5 agonist INT-777 increased energy expenditure, mitigated hepatic steatosis, and attenuated body weight gain by 15% [46]. In vitro, primary human brown adipocytes (prepared from subcutaneous fat and from the prevertebral BAT region) were treated with CDCA mitochondrial uncoupling and D2 expression [11].

Similar results were obtained with the use of a semi-synthetic derivative of CDCA—OCA. An enhanced brown adipocyte cell differentiation and up-regulated expression of the BAT-specific gene UCP1 were observed in C3H10T1/2 cells exposed to OCA in vitro. In the model of genetically programmed obesity in *db/db* mice, OCA increased the whole-body energy metabolism without altering food intake or physical activity and improved glucose homeostasis. The effect was associated with an enhanced BAT activity. OCA administration ameliorated body weight gain in *db/db* mice, treated with three different doses of OCA (7.5, 15.0, and 30.0 mg/kg daily) [12].

### 4.2. Clinical Studies

In view of encouraging experimental data, the impact of bile acids on energy expenditure, metabolism, and obesity under clinical conditions is gaining increasing attention. There is a positive correlation between the rise in circulating bile acids and the metabolic benefits seen after bariatric procedures [54,55,56,57,58]. It was proposed that increased levels of circulating bile acids via FXR-mediated signaling and alterations in microbiome are important aspects of effective bariatric surgery [58]. Others have shown that gastric bypass increases circulating bile acids and activates hepatic FXR [56]. The direct influence of bile acids on BAT metabolism was assessed in 12 healthy female subjects given CDCA (15 mg/kg) for 2 days. The therapy resulted in higher metabolic activity of BAT (by 5–6%) and increased whole-body energy expenditure [11].

OCA, developed as a treatment for various liver diseases, was approved in the USA and in the EU in 2016. It is permitted in the therapy of primary biliary cholangitis (PBC) in combination with ursodeoxycholic acid, or as monotherapy in patients unable to tolerate ursodeoxycholic acid [13,30]. In vitro, OCA affinity for FXR is 100-fold higher compared to endogenous bile acids, such as chenodeoxycholic acid [22,30]. Through selective activation of the FXRs on hepatocytes and enterocytes, OCA reduces the hepatotoxic potential of bile acids [59]. Typically, therapy of PBC starts at an initial dose of 5 mg once daily, which can be titrated to a maximum of 10 mg daily, if an adequate reduction in alkaline phosphatase (ALP) and/or bilirubin level within three months has not been achieved and a drug is well-tolerated [22].

Therapy with OCA has revealed beneficial results in treating other conditions such as metabolic-associated steatotic liver disease (MASLD), including its severe form, metabolic dysfunction-associated steatohepatitis—MASH (in daily doses from 10 to 40 mg), primary sclerosing cholangitis (5–10 mg daily), and type 2 diabetes (25–50 mg daily). However, follow-up studies are needed to assess and confirm the clinical usefulness of OCA in these conditions [59,60].

Hence, current evidence regarding the impact of OCA on body mass is primarily derived from populations with coexisting hepatic conditions [60,61,62]. In a double-blind, placebo-controlled trial, a 6-week OCA administration (N = 44; 25–50 mg daily) was performed in patients with MASLD and type 2 diabetes mellitus [60]. OCA stimulated insulin sensitivity by 24.5%, and evoked dose-related weight loss. The therapy was generally well-tolerated, and serious adverse effects have not been reported. Constipation and pruritus were incidental [46]. During the prospective, double-blind randomized FLINT trial (N = 141 participants with MASH; N = 142 placebo group), the administration of OCA (25 mg daily; 72 weeks) resulted in significant weight loss [61,62]. As compared to the placebo group, a higher proportion of patients treated with OCA manifested ≥ 2% weight loss. Importantly, during the follow-up, the rebound weight gain was not observed [61,62]. However, the beneficial effects on lipid and carbohydrate metabolism seen in placebo-treated patients who lost weight were absent in the OCA-treated cohort [62].

When evaluating the clinical relevance of FXR-mediated weight loss, it is essential to consider these outcomes in comparison with incretin-based therapies. While GLP-1 and dual GLP-1/GIP receptor agonists induce substantial weight reduction ranging from 15% to over 20%, FXR agonists such as OCA typically yield more modest results, mostly reaching 3–5%. However, the therapeutic value of FXR modulation should be viewed not only in isolated weight loss, but in its potent hepatoprotective and insulin-sensitizing properties, e.g., in patients with comorbid MASH. Unlike agents increasing satiety and reducing food intake, FXR ligands target the metabolic dysfunction, potentially offering a synergistic approach when used in combination with incretin mimetics.

Thus, the significance of clinical data reporting the effect of OCA on body mass requires careful assessment. First of all, a relatively small number of patients were enrolled in the studies. Furthermore, the duration and the doses of OCA varied. In addition, concomitant liver dysfunction may have obscured the impact of OCA on weight loss. The degree of weight loss is moderate, however, it is currently believed that the effect of applied therapy in the management of obesity, evaluated after a minimum of 12 weeks of treatment, should reach at least 5% reduction in body weight.

## 5. The Adverse Effects of OCA

The most common complaints in patients treated with OCA include pruritus, fatigue, abdominal pain and discomfort, and headache [13,22].

Pruritus can lead to treatment discontinuation in 1–10% of patients [30,61,63,64]. In POISE, a randomized, double-blind, placebo-controlled 12-month phase 3 trial performed in patients with PBC, pruritus was more common in the OCA group than in the placebo group (56–68% vs. 38%, respectively). Discontinuation rates due to this complaint were 1% and 10% in the OCA-5/10-mg and OCA-10-mg groups, respectively [65]. Pruritus was also the most common treatment-emergent adverse event in patients with MASH treated with OCA [61,63,64]. In REGENERATE, a randomized, controlled, phase 3 trial, pruritus was noted in 28–51% of participants (10 mg or 25 mg, respectively), compared to 19% in the placebo group. The incidence of pruritus was highest during the first 3 months of treatment. Withdrawal due to the complaint occurred in 1% of participants receiving 10 mg and 9% of those receiving 25 mg [64]. In the FLINT trial, pruritus was reported in 23% of treated patients (25 mg), compared to 6% in the placebo group [61]. While the incidence of pruritus is dose-dependent, most patients tolerate the drug well [59]. Rifampicin may be used to avoid pruritus [30]. Titration of the OCA dose from 5 to 10 mg, based on individual tolerance, may delay the onset of pruritus and reduce its severity [30].

Other adverse effects involve alterations in lipid metabolism, including reduced levels of HDL-cholesterol and triglycerides alongside an increase in LDL-cholesterol [22]. Lipid profile alterations typically manifest after several weeks of therapy and tend to stabilize or diminish over time. Notably, these metabolic changes are reversible upon discontinuation of treatment [61,65]. The CONTROL study demonstrated that OCA-evoked increases in LDL-cholesterol were ameliorated with atorvastatin, and the combination of both drugs was well-tolerated in patients with MASH [66]. Analysis of FLINT trial results revealed that OCA evokes a rise in small dense LDL particles, as well as an increase in small VLDL particles [67]. The long-term clinical significance of these findings remains to be fully elucidated, warranting a cautious approach. To our knowledge, no definitive correlation has yet been established between an altered lipid profile and an increased long-term cardiovascular risk [22]. Nevertheless, LDL cholesterol levels should be monitored during therapy, with pharmacological intervention initiated if necessary.

Serious or otherwise clinically significant liver-related adverse events in subjects with PBC, such as ascites and hepatic encephalopathy, are rare and have been reported mostly in subjects receiving high doses of OCA (higher than approved by the FDA) [13,30].

Notably, the reported adverse effects of OCA come from clinical studies with participants suffering from serious liver dysfunction (primary biliary cholangitis or steatohepatitis). Thus, their prevalence may be lower among patients with obesity without severe hepatic disease.

A comprehensive risk-benefit analysis is crucial when considering OCA and FXR agonists in the treatment of obesity. The modest efficacy in weight reduction must be weighed against a consistent profile of adverse effects, most notably dose-dependent pruritus and elevations in LDL cholesterol. While these lipid alterations are often transient or manageable with statins, they represent a significant hurdle for long-term adherence in a population already at high cardiovascular risk. However, there is no indication that OCA-induced lipid changes result in a substantial modification of cardiovascular risk scores or an increase in clinical cardiovascular adverse events. Therefore, characterizing the observed weight loss as promising requires caution; without evidence of long-term cardiovascular safety or a clear advantage over more potent weight-loss agents, the role of FXR agonists may be restricted to niche applications in metabolic liver diseases rather than broad-spectrum obesity management.

## 6. Clinical Trials with Other FXR Agonists

Over the last 10 years, orally available FXR agonists were developed and tested clinically, including e.g., tropifexor, cilofexor, vonafexor, nidufexor, or MET409 [66,68,69,70,71,72,73,74,75,76,77,78,79,80,81,82,83]. Vonafexor (EYP001a), tropifexor (LJN452), cilofexor (GS-9674), and nidufexor (LMB763) are synthetically modified variants of chenodeoxycholic acid. They belong to the second-generation, non-steroidal, non-bile acid, potent and highly selective FXR agonists [68,69,70,73,77,81,82].

The primary clinical goal of FXR agonists is the management of MASH, primary sclerosing cholangitis (PSC), PBC, and chronic hepatitis B [66,68,69,70,71,72,73,74,75,76,77,78,79,80,81,82,83]. Preliminary results reveal that FXR agonists improve fibrosis and steatosis, decrease liver fibrosis biomarkers, as well as improve biomarkers of cholestasis and cellular injury [66,68,69,70,71,72,73,74,75]. In patients with chronic hepatitis B, vonafexor demonstrated an anti-viral effect [76]. Furthermore, numerous studies indicate that FXR agonists evoke a reduction of body weight in different cohorts of patients [60,61,68,69,70,71,73,77,81,82].

LIVIFY trial, a double-blind phase IIa study, investigated the safety, tolerability, and efficacy of vonafexor in patients with suspected fibrotic MASH [69]. In part B of this trial, 96 patients were randomized. A single daily administration of oral vonafexor for 12 weeks of treatment led to a reduction in body weight. By week 12, a decrease in body weight of ≥3 kg was reported in 24.0% of patients in the VONA-100QD arm (vonafexor 100 mg/day), 30.8% of patients in the VONA-200QD arm (vonafexor 200 mg/day), compared to 15.6% of patients in the placebo arm. Body weight, waist circumference, waist-to-hip ratio, and waist-to-height ratio were also significantly reduced to a greater extent in the vonafexor treatment arms compared to placebo [69]. Weight loss with vonafexor was even higher than that observed after 72 weeks of treatment with OCA or cilofexor and nidufexor, and similar to that reported with tropifexor [62,68,69,70,73,77,81,82].

In a randomized, multicenter, double-blind, three-part adaptive design, phase 2 study FLIGHT-FX in patients with MASH, the safety and the efficacy of tropifexor (10–90 μg vs. 140 µg vs. 200 µg) were evaluated [73]. In parts A + B, 198 patients were randomized to receive tropifexor (10–90 μg) or placebo for 12 weeks. In part C, 152 patients were randomized to receive tropifexor 140 µg, tropifexor 200 µg, or placebo for 48 weeks. A dose-dependent reduction in body weight was observed with tropifexor 140 μg and 200 μg compared with placebo. At week 48, the LS mean decrease in body weight was greater in the tropifexor 140 μg- (−5.10 kg) and 200 μg- (−5.89 kg) groups versus placebo (−2.48 kg) [73].

The influence of tropifexor (TXR) on body mass was also evaluated in another study with patients who suffered from MASH [77]. TANDEM was a 48-week, phase 2b randomized, multicenter, double-blind study composed of 193 patients. A reduction in body weight of 2.5 kg was reached in patients treated with TXR 140 mg and persisted until week 48. Moreover, at week 48, the HOMA-IR score decreased in the TXR140 group only (change from baseline: −0.49) [77].

The ELIVATE study (randomized, double-blind, parallel-group, multicenter study), in which adult patients with MASH and liver fibrosis were enrolled, and the NEXSCOT study (a phase II, non-confirmatory, multicenter, open-label) revealed similar results [68,81]. In ELIVATE (N = 234), at week 48, a minimum 5% reduction of body weight was achieved by 52.2% of subjects given tropifexor (140 µg once daily) and by 80% of patients receiving tropifexor with licogliflozin 30 mg, compared with 12.5% of individuals in the placebo group, and 28.1% of those in the licogliflozin monotherapy group [68]. In the NEXSCOT study (N = 41), patients treated with tropifexor (200 µg once daily for 12 weeks) and an LYS006, an inhibitor of leukotriene A4 hydrolase, achieved at day 85 a mean 3.33 kg weight loss compared to a mean 0.54 kg in the monotherapy of LYS006 [81].

In a 24-week double-blind, placebo-controlled, phase 2 trial (140 patients with non-cirrhotic MASH), 100 mg, but not 30 mg of cilofexor daily, evoked a median weight loss of 1.4% [70].

In a phase 2 randomized patient-and-physician blinded, placebo-controlled, 24-week study (N = 83; participants with diabetic nephropathy, in combination with ACEI or ARB), a mean 0.61% decrease in body weight was achieved in the nidufexor group (50 mg once daily for 24 weeks), compared to a mean 0.55% weight gain in the placebo group. Similar beneficial changes were observed in BMI: mean 0.29% (BMI reduction) compared to mean 0.16% (BMI increase) [82,83].

Body weight loss was also achieved with MET-409, a novel fexaramine-derived non-bile acid FXR agonist [71]. In a 12-week, randomized, placebo-controlled study (48 participants with MASH), MET409 treatment resulted in body weight loss, with mean relative changes of −2.4% (80 mg) and −2.9% (50 mg) vs. −0.4% in placebo [71].

The adverse effects are mild to moderate in severity, with pruritus being the most frequent [68,69,70,71,73,74,75,76,77]. In some cases, pruritus caused the need to use anti-pruritic medications or to withhold treatment for short periods [62]. An increase in LDL-cholesterol has also been observed in some patients [62,70,71,75,77,78]. However, it is manageable with a low dose of statins [69,73]. In the LIVIFY study with vonafexor, a clinically relevant increase in LDL (>130 mg/dL) was observed in six out of 120 vonafexor-treated patients. In four of them statin treatment was initiated, and in the other two the existing statin doses were increased, leading to returning LDL levels to within the normal range [69]. In patients with MASH and hypertriglyceridemia, fenofibrate effectively mitigated increases in triglycerides evoked by cilofexor [79].

The metabolic outcomes of FXR signaling are clearly complex, beyond a simple agonist-equals-benefit. The evidence described above from tissue-specific knockout models reveals an intricate crosstalk where intestinal FXR antagonism and systemic FXR activation can, paradoxically, both lead to metabolic improvements under different physiological conditions. Thus, the net effect of FXR modulation seems to be determined by the baseline bile acid pool composition and the specific metabolic status of the host. Consequently, the translational success of FXR ligands may depend on identifying specific patient endotypes—such as those with primary bile acid malabsorption or distinct gut microbiota profiles—rather than applying a universal treatment strategy for obesity.

## 7. Conclusions and Future Directions

Considering the high economic cost, dramatically reduced quality of patients’ life, and severe complications of obesity, novel additional therapies are needed to strengthen the effect of currently approved drugs and to expand the panel of obesity management. OCA, as a semi-synthetic agonist of FXR and TGR5, is currently the only drug from the FXR-agonist group approved by the FDA for use in humans. The compound shows a therapeutic potential in animal models of obesity. The underlying mechanism seems to be linked with an enhanced energy expenditure in thermogenic tissues, leading to the subsequent weight loss. The preliminary data from the human studies suggest that OCA, similarly to other FXR agonists, may be effective in body weight reduction. When used in lower doses, it is well tolerated by patients. However, the obtained weight loss is rather moderate, and a single severe adverse effect was reported. The safety of the drug, especially its potential impact on lipid profile, should be evaluated carefully in large cohorts of patients. The assessment should take into account the age, gender, ethnic aspects, diet habits, and physical activity of enrolled individuals.

It is necessary to evaluate whether the co-administration of OCA with other anti-obesity drugs would be a more effective strategy than the administration of those drugs alone. It also seems important to assess whether OCA could be beneficial in preventing weight regain, commonly observed after initial weight loss. Finally, in view of preliminary data indicating that OCA may be an effective drug in patients with MASLD/MASH and type 2 diabetes, the two most common complications in patients with obesity, OCA could be valuable in the management of both the disease and its complications at the same time.

We propose a novel Bile Acid-Incretin Synergistic Framework for obesity management. We hypothesize that the lack of profound weight loss (≥10%) during monotherapy with FXR agonists is not a failure of potency, but a result of compensatory feedback loops, including the gut–brain axis. We suggest that future research should focus on the FXR-GLP-1 synergistic effect, where sub-therapeutic doses of FXR agonists are used to sensitize the hepatic and adipose insulin receptors prior to, or in conjunction with, GLP-1/GIP receptor agonism. Furthermore, we suggest that in MASH-adiposity subgroup, the primary goal of FXR modulation should not be total weight loss, but rather metabolic stabilization—the decoupling of adipose tissue expansion from hepatic lipotoxicity. This conceptual change from weight-centric to organ-protective outcomes offers a testable hypothesis for future clinical trial designs, prioritizing patient stratification rather than using BMI alone.

## Data Availability

No new data were created or analyzed in this study.

## Abbreviations

The following abbreviations are used in this manuscript:

ACEI	angiotensin-converting enzyme inhibitors
ARB	angiotensin II receptor blockers
ALP	alkaline phosphatase
BAT	brown adipose tissue
BA	bile acid
BMI	body mass index
CA	cholic acid
CDCA	chenodeoxycholic acid
D2	type 2 iodothyronine deiodinase
DCA	deoxycholic acid
GI	gastrointestinal system
GLP-1	glucagon-like peptide-1
LCA	lithocholic acid
LDL-cholesterol	low-density lipoprotein cholesterol
FGF-19	fibroblast growth factor 19
FGFR-4	fibroblast growth factor receptor 4
FXR	farnesoid X receptor
MAFLD	metabolic-associated fatty liver disease
MASH	metabolic-associated fatty liver disease steatohepatitis
OCA	obeticholic acid
PBC	primary biliary cholangitis
PSC	primary sclerosing cholangitis
PPARα and PPARγ	peroxisome proliferator-activated receptor-alpha and gamma
SGLT1/SGLT2	sodium/glucose cotransporter 1/2
UCP1	uncoupling protein 1
TGR5	Gαs-protein-coupled bile acid receptor
